# A New Mouse Model to Study Acquired Lymphedema

**DOI:** 10.1371/journal.pmed.0030264

**Published:** 2006-07-18

**Authors:** Martin Schneider, Annelii Ny, Carmen Ruiz de Almodovar, Peter Carmeliet

## Abstract

Schneider and colleagues say that the new model, published in *PLoS Medicine,* promises to increase our understanding of lymphedema and hopefully accelerate the development and testing of new treatments.

More than 1 million women worldwide develop breast cancer every year [ 1]. For many of them, surgical removal of the breast remains the first line of treatment for this potentially deadly disease. Because breast cancer cells spread via lymph vessels, at the time that the breast is resected, locally involved lymph nodes in the armpit and part of the axillary lymphatic network are usually also removed. Consequently, normal drainage of lymph is often interrupted, causing swelling of the affected arm due to lymph accumulation—a condition termed lymphedema. Acquired lymphedema in humans may also result from irradiation, trauma, or (parasitic) infection.

While the swelling causes discomfort and disability, an even greater health threat lies hidden in the structural and functional changes inside the chronically lymphedemic tissue. Adipocytes, keratinocytes, and fibroblasts accumulate, transforming the initially soft swollen tissue into a hard fibrotic mass with fatty degeneration and a stiff, thickened skin [ 2–4]. In addition, there is reduced trafficking of antigen-presenting and other immune cells to the lymph nodes, and so these cells are less likely to be able to perform their immune surveillance function to defend the host against foreign antigens. As a result, the tissues affected by lymphedema are prone to persistent inflammation and infection [ 2, 4]. The increased interstitial tissue pressure may collapse the veins, further aggravating the condition and in severe cases even necessitating amputation.

Almost unimaginably, close to 400 years after the discovery of lymph vessels, there is still no cure for lymphedema, and current medical practice still relies on ancient procedures, such as manual lymph drainage via massage. A better knowledge of the molecular cues underlying the abnormalities that characterize the inflammatory tissue response to lymph stagnation is thus urgently needed to provide novel perspectives for lymphedema treatment. However, research progress in the field has been hampered by the lack of suitable experimental animal models. The new mouse model of acquired lymphatic insufficiency reported by Tabibiazar et al. in *PLoS Medicine* [ 5] may help to overcome some of these obstacles.

There is still no cure for lymphedema, and current medical practice relies on ancient procedures.

## The New Mouse Model

How did the authors develop and characterize the model? Microsurgical ablation of the lymph vessels in the tail of the mouse resulted in lymph stagnation, lymph vessel dilation (with a marked increase in tail volume), accumulation of fibroblasts, fat, and skin cells, impaired clearance of immune cells from the tail, and profound accumulation of inflammatory cells. This novel model thus closely recapitulates several hallmark features of acquired lymphedema in humans.

The model also differs from the human disease in certain aspects, however. For instance, a mouse tail lacks lymph nodes, which may play a critical role in fostering the immune response and inflammation in the edematous tissue in human lymphedema [ 6]. It remains to be assessed whether the absence of lymph nodes influences the tissue response to lymph stasis in the mouse tail. The hydrodynamic and cellular mechanisms and the rate of lymph drainage in a horizontally positioned mouse tail also differ from those in a supine human limb, in which positional changes and muscle contractions determine lymph drainage [ 7]. It is unclear to what extent these differences influence the disease course and severity. Moreover, it will be interesting to characterize the long-term chronic structural changes in the lymphedemic mouse tail.

Interestingly, not only were lymph vessels dilated in the mouse tail model, but there were also 10-fold more lymphatic vessels in the lymphedemic tail than in control tails. It remains to be determined whether this increase in the number of lymphatic vessels in the lymphedemic tissue is a peculiarity of the mouse tail model, as very little information about lymphatic hyperplasia in humans with lymphedema is available. While the precise reason for this increased lymphatic vessel density remains unclear, it is conceivable that the large accumulation of inflammatory cells and the release of inflammatory cytokines may have stimulated lymphangiogenesis [ 8, 9]. Another unresolved issue is to what extent inflammatory cells are merely trapped in the edematous tissue as silent bystanders or, instead, actively determine disease progression. The role of inflammation in the tissue response to lymphedema in this mouse model could be readily tested by using immunocompromised mice or knockout mice lacking specific inflammatory mediators, or by treating mice with anti-inflammatory agents.

## Comparison with Previous Models

To what extent does this new model differ from existing ones? Is the new model any better?

Several models of lymphedema have been previously developed. Some were developed via surgical ablation or irradiation of the lymphatics in the rabbit ear or mouse tail [ 10–13], others via inactivation or mutation of lymphangiogenic genes in mice, such as the *Chy* mice carrying a heterozygous *Vegfr3* mutation [ 14]. These models have been generally used to study the physiological regulation of lymph flow and to assess the therapeutic potential of VEGF-C to stimulate lymphatic revascularization.

However, apart from the *Prox1 ^+/−^* mouse, which accumulates fat as a consequence of lymphatic vascular leakage [ 15], none of these models has been used to study the chronic secondary tissue changes in response to lymphedema. Thus, unlike the previous models, Tabibiazar and colleagues' new mouse model of acquired lymphedema, in combination with powerful mouse genetics, offers exciting opportunities to dissect the molecular basis of the response to lymphedema. In addition, this new model should be valuable to evaluate the therapeutic potential of novel anti-lymphedema drugs.

## Clinical Implications

One of the reasons why treatment of lymphedema is still in its infancy relates to our lack of understanding of the changes induced by lymph stagnation. Previous studies primarily focused on identifying the gene programs switched on in lymphatic endothelial cells in response to lymphangiogenic stimuli [ 16, 17]. However, more cell types participate in the complex response to lymph stasis than lymphatic endothelial cells alone. For instance, chyle itself stimulates adipogenesis [ 15], and it is conceivable that yet unidentified chemokines and growth factors in lymph affect other cell types in edematous tissues as well.

As Tabibiazar et al. show, their new model provides opportunities to identify sets of genes previously unsuspected to be involved in acquired lymphedema. Their current analysis has already identified genes regulating inflammation, immunity, complement activation, fibrosis, oxidative stress, and angiogenesis. Obviously, the functional significance of the current gene profile will need to be further validated. Other small animal models, such as the recently generated *Xenopus laevis* tadpole model of lymphangiogenesis, may accelerate the functional screening of such candidates [ 18]. Overall, the new mouse model of acquired lymphedema promises to increase our understanding of this largely unexplained disease, and hopefully accelerate the development and testing of new treatments for this devastating disorder.
